# CRISPR/Cas9-mediated reporter knock-in in mouse haploid embryonic stem cells

**DOI:** 10.1038/srep10710

**Published:** 2015-06-03

**Authors:** Yasuyoshi Kimura, Masaaki Oda, Tsunetoshi Nakatani, Yoichi Sekita, Asun Monfort, Anton Wutz, Hideki Mochizuki, Toru Nakano

**Affiliations:** 1 Department of Pathology; 2 Department of Neurology, Graduate School of Medicine; 3 Graduate School of Frontier Biosciences, Osaka University, Osaka, Japan; 4 Department of Biosciences, Kitasato University School of Science, Kanagawa, Japan; 5 Institute of Molecular Health Sciences, Swiss Federal Institute of Technology, ETH Zürich, Hönggerberg, 8049 Zürich, Switzerland; 6 JST, CREST.

## Abstract

Mouse parthenogenetic haploid embryonic stem cells (ESCs) are pluripotent cells generated from chemically activated oocytes. Haploid ESCs provide an opportunity to study the effect of genetic alterations because of their hemizygotic characteristics. However, their further application for the selection of unique phenotypes remains limited since ideal reporters to monitor biological processes such as cell differentiation are missing. Here, we report the application of CRISPR/Cas9-mediated knock-in of a reporter cassette, which does not disrupt endogenous target genes in mouse haploid ESCs. We first validated the system by inserting the P2A-Venus reporter cassette into the housekeeping gene locus. In addition to the conventional strategy using the Cas9 nuclease, we employed the Cas9 nickase and truncated sgRNAs to reduce off-target mutagenesis. These strategies induce targeted insertions with an efficiency that correlated with sgRNA guiding activity. We also engineered the neural marker gene *Sox1* locus and verified the precise insertion of the P2A-Venus reporter cassette and its functionality by monitoring neural differentiation. Our data demonstrate the successful application of the CRISPR/Cas9-mediated knock-in system for establishing haploid knock-in ESC lines carrying gene specific reporters. Genetically modified haploid ESCs have potential for applications in forward genetic screening of developmental pathways.

Haploid model organisms are powerful tools for exploring gene functions because they possess a single set of chromosomes. Hemizygosity of all genes allows for efficient disruption of gene function and unveils recessive phenotypes^1^. In mammals, near-haploid human tumour cells are primarily used in genetic screening for the identification of factors that mediate susceptibility to pathogens, drugs, or toxicities^1^,^2^. Recently, parthenogenetic and androgenetic haploid embryonic stem cells (ESCs) have been generated from mouse embryos^3^,^4^,^5^,^6^ and are considered an excellent tool for exploring the function of specific genes.

Mouse haploid ESCs have a transcriptional profile similar to their diploid counterparts and maintain the capacity for cellular differentiation and germline chimera production^3^,^4^,^5^,^6^,^7^. It has been shown that mouse haploid ESCs are also applicable to genetic screens of the mismatch repair pathway and to explore susceptibility to toxicities^3^,^4^,^8^. In addition to screens targeting these “life-or-death matter” pathways, one recent study identified novel regulators involving exit from the pluripotent state. This study employed the haploid ESC lines derived from a transgenic mouse strain expressing a fluorescent marker inserted under the control of the *Zfp42* gene promoter^9^. However, such applications remain limited because it could be time-consuming to establish haploid cell lines from transgenic mice with ideal reporters. Moreover, the reporter cassette would need to be inserted without disrupting the target gene to avoid recessive loss-of-function mutations in the haploid genome.

The clustered regularly interspaced short palindromic repeats (CRISPR)/CRISPR-associated protein (Cas) system has emerged as a simple and efficient genome editing method *in vitro* and *in vivo*^10^,^11^,^12^,^13^. The Cas9 nuclease from *Streptococcus pyogenes* is directed to specific genomic loci through an engineered chimeric single-guide RNA (sgRNA) containing 20 nucleotides (nt) complementary to the target DNA sequence^10^. The guided Cas9 introduces a double-strand break (DSB), which is repaired by non-homologous end-joining (NHEJ) or homology-directed repair (HDR) pathways (Supplementary Fig. S1). NHEJ frequently results in deletion/insertion mutations of the target region, while HDR mediates targeted insertion of a donor template with homologous sequences to the DSB-flanking regions^14^,^15^,^16^,^17^,^18^.

Several approaches based on the CRISPR/Cas9 system have recently been developed to reduce undesired off-target mutagenesis; for example, the use of the Cas9 nickase and truncated sgRNAs. The Cas9-D10A mutant nickase (Cas9n), in which aspartic acid at codon 10 is altered to alanine in the RuvCI nuclease domain, induces a nick in the genome, in contrast to DSB induction by Cas9^10^,^19^. Double nicking by Cas9n with a pair of offset sgRNAs induces DSBs by reducing off-target mutagenesis^20^,^21^,^22^,^23^ since individual nicks are preferably repaired by high fidelity base excision repair^24^. It has also been demonstrated that Cas9n-induced individual nicks are sufficient to trigger HDR in the human genome^14^,^20^,^25^. The use of truncated sgRNAs with 17–19 nt of target complementarity are known to enhance base-pairing specificity and reduce off-target activity^26^.

In this study, we applied the CRISPR/Cas9 system to insert a reporter cassette into mouse haploid ESCs in culture. We established haploid ESC lines expressing Venus concomitant with a housekeeping gene *Actb (actin, beta*) or a neural specific gene *Sox1* (*Sex determining region Y-box1*) and verified the usefulness of the inserted reporter. Our data demonstrate the feasibility of using recent CRISPR/Cas9 techniques for engineering the haploid ES cell genome that could contribute to extending the range of future genetic screens.

## Results

### CRISPR/Cas9-mediated reporter knock-in at the *Actb* locus in mouse haploid ESCs

**Figure 1:**
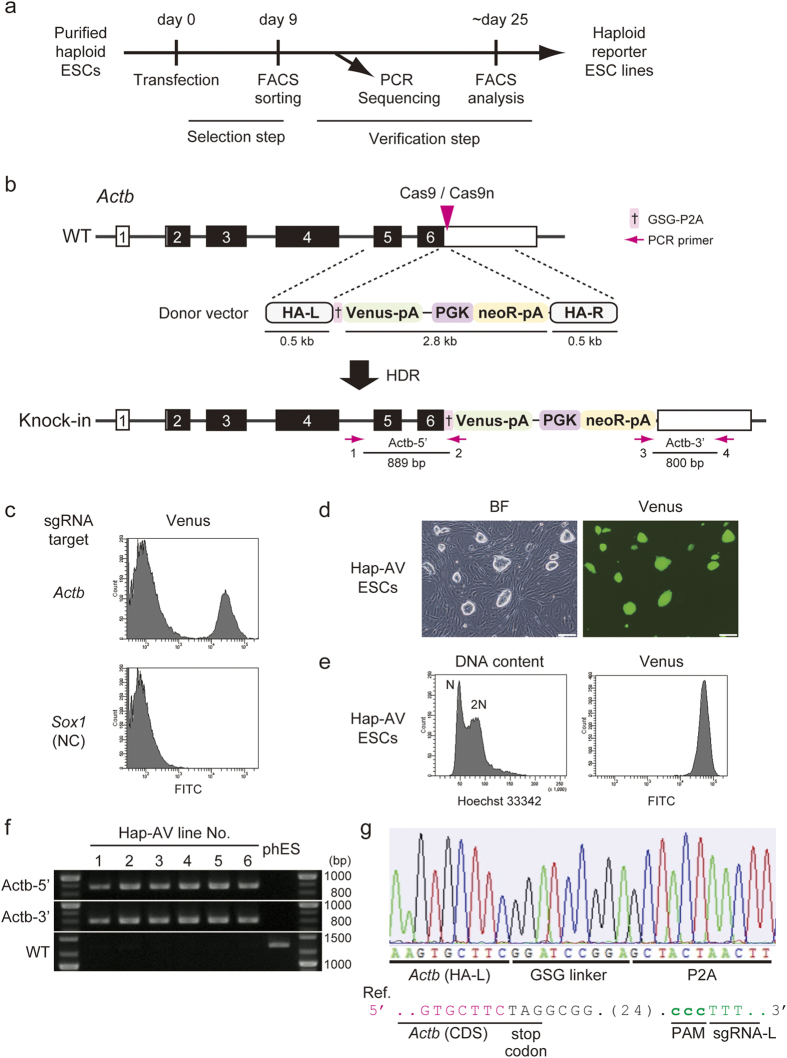
CRISPR/Cas9-mediated reporter knock-in at the Actb locus in mouse haploid ESCs. (**a**) Experimental scheme to establish haploid reporter ESC lines in culture. (**b**) Schematic illustration of the strategy to insert a P2A-Venus reporter cassette into the *Actb* locus. The upper diagram shows the *Actb* wild-type (WT) allele, which contains coding sequences (CDS) shown in black boxes and untranslated regions (UTR) shown in white boxes. The position of the single-guide RNA (sgRNA) target is indicated by a magenta arrowhead. The middle diagram shows the donor vector that contains the left and right homologous arms (HA-L and HA-R) and the Venus and neomycin resistance gene (neoR) driven by the PGK promoter. The bottom diagram represents the knock-in allele. Magenta arrows are PCR primers used to check the knock-in allele. (**c**) FACS analysis of Venus fluorescence after knock-in of the reporter cassette to the *Actb* locus. FACS analysis was performed 9 days after co-transfection of the donor vectors and the pX330 plasmids carrying a sgRNA targeting *Actb* (top) or *Sox1* (bottom, Negative control) in haploid ESCs. (**d**) Bright field (BF) and fluorescence images of a Hap-AV cell line. A representative line (line No. 1) is shown. Scale bar, 200 μm. (**e**) Characterisation of the Hap-AV cell line using FACS. FACS analysis of the DNA content (left) and Venus fluorescence (right) are shown. (**f**) Targeted knock-in of the reporter cassette detected by genomic PCR in the Hap-AV lines. Representative 6 Hap-AV lines (No. 1–6) and their parental haploid ESC line (phES) are shown in the cropped gel images. Full length gels are presented in Supplementary Fig. S4. Primers and expected fragment sizes of each PCR amplicon; Actb-5’ = primer 1 and 2 (889 bp), Actb-3’ = primer 3 and 4 (800 bp), WT = primer 1 and 4 (1336 bp). (**g**) Sequence of the knock-in allele in the Hap-AV cells. The sequence of the WT allele is indicated at the bottom as a reference. PAM, protospacer adjacent motif.

We established mouse parthenogenetic haploid ESCs, as described previously^3^, since long-term culture of ESCs is associated with chromosomal abnormalities and epigenetic instability^27^,^28^,^29^. A total of 51 blastocysts were developed from 115 activated oocytes, and 25 ESC lines were established (Supplementary Fig. S2). Eight randomly selected ESC lines were analysed for DNA content and all of the analysed lines contained the haploid populations (Supplementary Fig. S2). We maintained the haploid ESCs purified using periodic flow cell sorting of their haploid 1n peaks to eliminate self-diploidised cells in culture^3^,^9^,^30^. Purified haploid ESCs were then used to establish the reporter cell lines (Fig. 1a).

A reporter cassette in a donor vector was designed to fuse with the coding region (CDS) of a target gene via a P2A sequence that encodes a small self-cleaving linker peptide^31^,^32^ (Supplementary Fig. S1). For this purpose, the 3’ untranslated region (UTR) of the target gene was selected as a CRISPR/Cas9 target site and approximately 0.5 kb of the left and right sides of the target site were used for the homologous arms (HAs) in the donor vector. The left HA did not contain the stop codon of the target gene. Therefore, homologous recombination of the donor vector triggered by CRISPR/Cas9-mediated cleavage should lead to concordant expression of the endogenous target gene and the reporter transgene.

We chose the *Actb* locus to examine whether CRISPR/Cas9-mediated knock-in of the reporter cassette functioned in the mouse haploid ESCs (Fig. 1b). The CRISPR/Cas9 target site was designed in the 3’UTR of the last exon (exon 6) of the *Actb* gene. Synthesised oligonucleotides for sgRNA targeting sequences were cloned into the pX330 plasmid, in which both Cas9 and an inserted sgRNA were expressed^14^,^33^. The on-target excision efficiency of sgRNAs was evaluated using the single-strand annealing (SSA) assay based on reconstitution of reporter gene expression (Supplementary Fig. S3). The pCAG-EGxxFP plasmid for the SSA assay contained 5’ and 3’ EGFP fragments sharing 482 bp as a target plasmid^34^. Cas9 was recruited to the target sequence by a functional sgRNA and cut the genomic target fragment cloned into the EGxxFP fragment, leading to reconstitution of the *EGFP* gene via HDR. The donor vector targeting *Actb* contained a reporter cassette with a fluorescent marker Venus following P2A (Fig. 1b). A neomycin resistance gene (neoR) driven by the phosphoglycerate kinase enhancer/promoter (PGKp) was added to facilitate isolating the reporter-integrated clones. Thus, targeted insertion of this donor vector via HDR should result in G418 resistance and concordant expression of Actb and Venus.

Mouse haploid ESCs were co-transfected with circular donor vectors and pX330 plasmids, and cultured under G418 selection. Venus-positive haploid G1 cells from independent culture wells were sorted using a fluorescence-activated cell sorter (FACS) (Fig. 1c), and were subsequently cultured on inactivated mouse embryonic fibroblast (MEF) cells at a low density. Individual reporter ESC lines were obtained from single clones (Fig. 1d,e). All analysed lines (18 of 18 Venus-positive ESC lines) possessed the knock-in allele at the *Actb* locus without any sequence error (Fig. 1f,g and Supplementary Fig. S4). Six of eighteen (33%) Venus-positive ESC lines maintained the haploid proportion, whereas 12 of 18 (67%) cell lines were diplodised during the selection process.

We constructed another reporter cassette to perform the selection step more efficiently. A hygromycin resistance gene (hygroR) was conjugated after Venus via a T2A sequence that encodes another self-cleaving small peptide (P2A-Venus-T2A-hygroR). Almost all hygromycin-resistant cells (97.6% in haploid ESCs and 96.4% in diploid ESCs) were Venus-positive (Supplementary Fig. S5). These results demonstrated that our donor vectors could be used for CRISPR/Cas9-mediated reporter knock-in without disrupting the target gene in haploid ESCs.

### Knock-in efficiency varies with different combinations of Cas9 types and sgRNA length

Next, we examined the knock-in efficiency of the reporter cassette at the *Actb* locus under various combinations of Cas9 types and sgRNA length, which are known to reduce off-target mutagenesis^14^,^20^,^21^,^22^,^23^,^25^,^26^. Two types of sgRNA; namely, full-length (20 nt) or truncated (17 nt), were designed in the 3’UTR of *Actb* and co-expressed with Cas9 or Cas9n for the SSA assay (Supplementary Fig. S3). EGFP reconstitution was more frequently detected in the paired Cas9n using off-set sgRNAs than for Cas9. We then examined the knock-in efficiency of the donor vector using these combinations of the Cas9 types and sgRNAs in haploid ESCs (Table 1).

The knock-in efficiency in each combination was validated based on the ratios of Venus-positive cells after G418 selection (Combination Nos.1–9 in Table 1 and Supplementary Fig. S6, hereinafter referred to as C-Nos.1–9). Diploid J1 ESCs were also used as a reference for knock-in efficiency. CRISPR/Cas9-mediated knock-in was successful in both haploid and diploid ESCs when sgRNA(s) and HAs of the donor vector targeted *Actb* (C-Nos.1–6 in Table 1 and Supplementary Fig. S6). The double-nicking strategy mediated by the paired Cas9n (C-Nos.3 and 4) showed higher targeting efficiency than the strategy with Cas9 (C-Nos.1 and 2) or Cas9n alone (C-Nos.5 and 6) in haploid ESCs. There were no or only a few Venus positive cells when either sgRNAs or HAs were used that were not matched to the target (C-Nos. 7 and 8) or when the donor vector alone was transfected (C-No. 9). The knock-in efficiency with the paired Cas9n (C-Nos.3, 4) in diploid ESCs was also high, albeit the efficiency observed at C-Nos.1–4 was about half that in haploid ESCs. These results indicated that DSBs or individual nicks induced by the CRISPR/Cas9 system did trigger homologous recombination in mouse haploid ESCs with a practicable efficiency as it did in diploid ESCs.

### Generation of a Sox1-reporter in haploid ESCs using the CRISPR/Cas9 strategy

**Figure 2:**
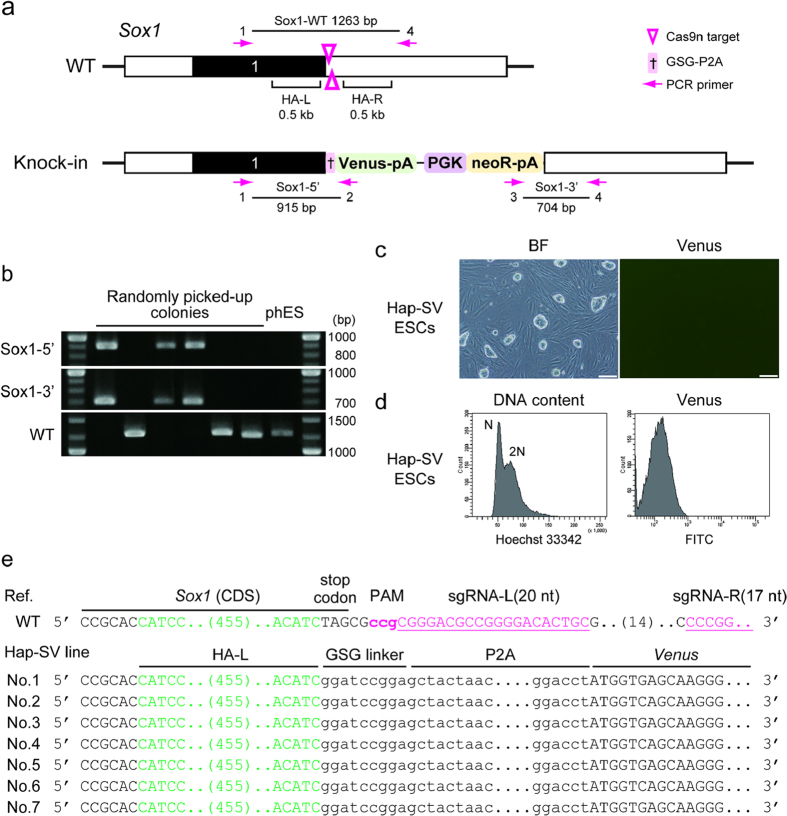
Generation of a Sox1-reporter in haploid ESCs using the CRISPR/Cas9 strategy. (**a**) Schematic illustration of the WT and knock-in alleles at the *Sox1* locus. Black and white boxes indicate CDS and UTR, respectively. The sgRNA target sites for double nicking are indicated by magenta arrowheads. The left and right homologous arms are indicated as HA-L and HA-R, respectively. PCR primers used for Fig. 2b are shown as magenta arrows. (**b**) Targeted knock-in of the reporter cassette detected by genomic PCR in randomly selected neomycin-resistant haploid colonies. Their parental haploid ESC line (phES) is shown as a control. The cropped gel images are displayed and full length gels are presented in Supplementary Fig. S4. (**c**) Bright field (BF) and fluorescence images of the established Hap-SV line. Representative Hap-SV cells (line No. 1) were shown. Scale bar, 200 μm. (**d**) Characterisation of the Hap-SV cell line. FACS analysis of the DNA content (left) and Venus fluorescence (right) are shown. (**e**) Sequences of the knock-in allele in 7 Hap-SV cell lines. The sequence of the WT allele is indicated at the top as a reference. PAM, protospacer adjacent motif.

To demonstrate the usefulness of our CRISPR/Cas9 strategy, we engineered a construct for the earliest neural precursor gene *Sox1* locus^35^,^36^,^37^ and established mouse haploid ESC lines carrying a Sox1-P2A-Venus reporter (Hap-SV) to monitor neural differentiation (Fig. 2a). A previous reporter at the *Sox1* locus has been inserted behind its promoter^36^, which would cause a loss of the endogenous *Sox1* in haploid cells. To circumvent this problem we designed a knock-in strategy for a reporter cassette in this study that results in a gene fusion facilitating the concordant expression of Sox1 and Venus, thereby preventing the loss of Sox1 function (Fig. 2a). The *Sox1* gene consists of a single exon and the 3’UTR was selected for the CRISPR/Cas9 target site, similar to the strategy used for the *Actb* locus. We adopted the double-nicking strategy using the paired Cas9n to trigger HDR since this strategy showed the highest knock-in efficiency in the case of *Actb*. An offset sgRNA pair (left-20 nt and right-17 nt) used for further experiments was validated using the SSA assay (Supplementary Fig. S3).

The Venus reporter driven by Sox1 activity was detectable along the neural differentiation, but not at the ESC state. Therefore, knock-in of the reporter cassette in neomycin resistant ESC lines was examined based on PCR analysis of the genomic DNA (Fig. 2b,c and Supplementary Fig. S4). Twelve of twenty-six (46%) ESC lines possessed the knock-in allele at the *Sox1* locus (Table 1). Seven of twelve (58%) reporter-integrated ESC lines maintained the haploid genome even after the selection process and were used as Hap-SV lines (Fig. 2c,d). All Hap-SV cell lines possessed the knock-in allele at the *Sox1* locus without any sequence error (Fig. 2e and Supplementary Fig. S7).

### Neural differentiation of the haploid ESCs carrying the Sox1-P2A-Venus allele

**Figure 3:**
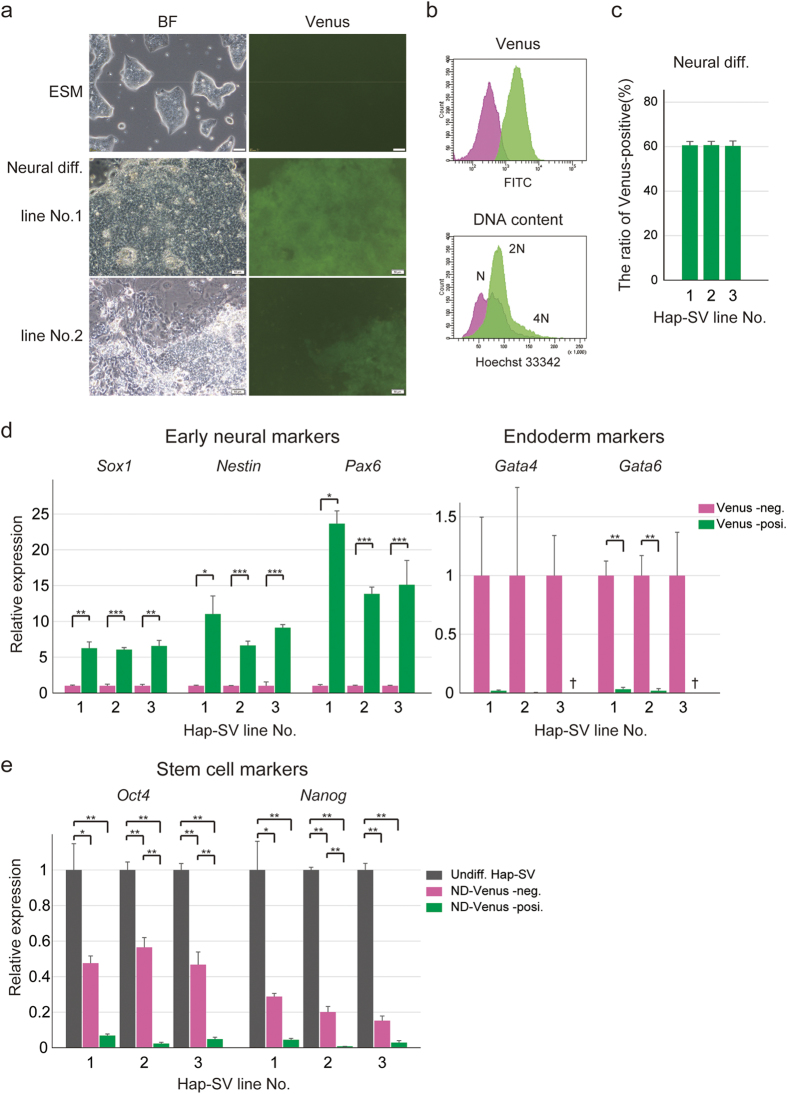
Neural differentiation of haploid ESCs carrying the Sox1-P2A-Venus reporter. (**a**) Morphology and Venus fluorescence images of the differentiated cells in N2B27 culture from Hap-SV ESCs. Top shows Hap-SV ESCs before differentiation. BF, bright field; scale bar, 50 μm. (**b**) Venus fluorescence and DNA content analysis of the differentiated neural cells 9 days after the differentiation induction of Hap-SV cells. The Venus-negative population is shown in magenta and the Venus-positive population is shown in green. (**c**) The Venus-positive ratio in differentiated Hap-SV lines. FACS analysis was performed at day 9 after differentiation induction in N2B27 culture. Data from three Hap-SV lines are shown as the means ± SEM (n = 3). (**d**) Expression of differentiation marker genes in differentiated Hap-SV cells. RT-qPCR was performed 9 days after the differentiation induction in N2B27 culture. Venus-negative (magenta) and Venus-positive (green) sorted populations were analysed. Relative expression levels were normalised to the *Gapdh* expression level. Values in Venus-negative populations were set to 1. Data are shown as the means ± SEM (n = 3 for each line). Statistical significance was determined using Student’s *t*-test. ^*^, *p* < 0.05; ^**^, *p* < 0.01; ^***^, *p* < 0.0001. †, not detected. (**e**) Expression of stem cell marker genes in the undifferentiated and differentiated Hap-SV cells. Differentiated Hap-SV cells were collected at day 9 after differentiation in N2B27 culture. Values in undifferentiated Hap-SV ESCs were set to 1. Statistical significance was determined using Tukey’s multiple comparison analysis at the 5% (^*^) and 1% (^**^) significance level. Undiff., undifferentiated; ND, neurally differentiated.

To evaluate the functional capability of the Sox1-P2A-Venus reporter, *in vitro* neural differentiation was induced in Hap-SV cells by adherent monoculture in the N2B27 medium, which drives more than 60% of diploid ESCs into the neural lineage^37^,^38^. Three independent Hap-SV ESC lines and their parent haploid ESCs were more vulnerable to the N2B27 condition than diploid ESCs and retained dome-like appearance in part. After 5-6 days, the induced cells gradually showed neuronal morphology as observed in the diploid cells^37^,^38^, and then Venus positive cells became detectable. Venus fluorescence became more evident around day 9-10 (Fig. 3a) when *Sox1* mRNA was highly expressed along the neural differentiation. Neural differentiation of Hap-SV cells was also confirmed by Western blot analysis (Supplementary Fig. S8) and immunostaining (Supplementary Fig. S9). In addition, FACS analysis showed that about 60% of the cells of all lines differentiated to Venus-positive cells (Fig. 3b,c). DNA content analysis revealed that all Venus-positive cells have been diploidised and Venus-negative cells retained the haploid DNA contents (Fig. 3b). Reverse transcription quantitative PCR (RT-qPCR) analysis confirmed the expression of marker genes for Venus-positive populations: early neural marker genes *Sox1*, *Nestin*, and *Pax6* were highly expressed in Venus-positive populations. The expression of endoderm marker genes *GATA4* and *GATA6* was undetectable or quite low (Fig. 3d). Stem cell marker genes *Oct4* (also known as *Pou5f1*) and *Nanog* were significantly down-regulated in both Venus-positive and -negative populations, albeit down-regulation was more prominent in Venus-positive populations (Fig. 3e). The results of stem cell marker and DNA content analyses were consistent with previous reports describing that non-neural populations in neural differentiation contained undifferentiated cells^37^ and neural differentiation of haploid ESCs was accompanied by diploidisation^3^,^7^. We also evaluated this Sox1-P2A-Venus reporter using another *in vitro* neural differentiation method; namely, serum-free floating culture of embryoid body-like aggregate (SFEB)^39^. We verified that Venus-positive cells differentiated by SFEB possessed early neural properties, similar to those in N2B27 adherent monoculture (Supplementary Fig. S10). We conclude that our new Sox1-P2A-Venus reporter is useful for monitoring the entry of pluripotent haploid cells into neuroectodermal differentiation.

## Discussion

In this study, we performed CRISPR/Cas9-mediated knock-in of the reporter to monitor endogenous target gene expression without disrupting the target gene in mouse haploid ESCs. The usefulness of the Sox1-P2A-Venus reporter for monitoring neural differentiation was demonstrated based on the emerging ratio and gene expression profiles of Venus-positive cells. Off-target mutagenesis is an important issue^11^,^21^,^23^,^25^,^40^,^41^,^42^,^43^,^44^ in the CRISPR/Cas9 technology, and methods for reducing off-target mutagenesis have recently been developed^14^,^20^,^21^,^22^,^23^,^25^,^26^. We validated these methods and demonstrated that the Cas9 nickase and/or truncated sgRNAs could be used to trigger HDR in mouse haploid ESCs (Table 1 and Supplementary Fig. S6). In particular, single nick-mediated HDR could be a valid choice for diminishing undesired off-target mutagenesis, because individual nicks are predominantly repaired by high-fidelity HDR or BER^24^,^45^. One noteworthy development of the CRISPR/Cas9 mediated knock-in technique is the CRIS-PITCh (Precise Integration into Target Chromosome) strategy which utilizes microhomology-mediated end-joining (MMEJ)^46^. In CRIS-PITCh, three sgRNAs with Cas9 nuclease are required for cassette integration and donor vectors are linearized in the transduced cells, which could give rise to off-target mutagenesis and random integration of linearized vectors. CRIS-PITCh, however, has the advantage of enabling targeted insertion of DNA fragments in various cells and organisms including those with low HDR activity, since MMEJ repair and HDR is active during G1/early S phase and late S/G2 phase in the cell cycle, respectively^47^. We assume that CRIS-PITCh would function in haploid cells and facilitate genome engineering of haploid cells with our knock-in strategy.

The knock-in efficiency in our study differed between the *Actb* and *Sox1* loci, as well as between the *Actb* locus depending on the combination of Cas9 types and sgRNAs (Table 1). This could be explained by the difference in guiding activity of designed sgRNAs since the knock-in efficiency in ESCs tended to be consistent with on-target cleavage activity observed in the SSA assay (Table 1 and Supplementary Fig. S3). In our study, GC contents of sgRNAs influenced the results of the SSA assay: 17–20 nt sgRNAs with more than 7 GCs showed high cleavage activity, although too high of a GC% might lead to loss of activity (Supplementary Table S1 and unpublished data). This result supports the recent report showing that genome-engineering efficiency correlates with the sgRNA GC content 6 nt adjacent to the protospacer adjacent motif (PAM) in *drosophila*^48^. It is also notable that double nicking with the paired Cas9n showed higher cleavage efficiency than the individual Cas9 with the same sgRNA (Supplementary Table S2 and unpublished data). Considering that Cas9 binding events occur more frequently in open chromatin regions than closed regions^43^,^44^, other factors, such as the chromosomal accessibility of target regions, also influence the knock-in efficiency. Taken together, the use of validated highly functional and specific sgRNAs is important to increase the success rate of CRISPR/Cas9-mediated genome engineering.

The knock-in efficiency at the *Actb* locus induced by Cas9 and the paired Cas9n was significantly higher in haploid ESCs than in diploid J1 ESCs (C-Nos.1–4 in Table 1 and Supplementary Fig. S6). In contrast, there was no significant difference between haploid and diploid in the knock-in efficiency induced by the single Cas9n (C-Nos.5 and 6 in Table 1 and Supplementary Fig. S6). DSBs by Cas9 or the paired Cas9n were induced adjacent to the *Actb* CDS, some of which may result in *Actb* disruption via NHEJ without the reporter knock-in. The hemizygotic haploid ESCs would die in this situation more frequently than diploid because acute *Actb* loss results in a growth defect and cell death of cultured cells^49^. This would lead to a reduced number of surviving colonies and the apparent increase in knock-in efficiency in haploid ESCs. On the other hand, a single nick at the *Actb* locus (C-Nos.5 and 6) would be repaired mainly by HDR or BER; hence, Actb function would be preserved in haploid ESCs, similar to diploid cells. Reporter-integrated ESCs would be isolated more effectively in haploid ESCs when a target gene is essential for ESC survival. We consider this a general rule of our strategy, as well as for the *Actb* gene.

Mouse haploid reporter ESCs have been established from a transgenic mouse strain in the previous report^9^. This strategy would be advantageous provided that the mouse reporters have already been validated^1^. However, such transgenic mice may not always be available. Besides, transgenic mice with reporters disrupting the endogenous genes would not be appropriate for haploid ESCs. In such cases, the CRISPR/Cas9-mediated knock-in strategy will provide a practicable and rapid approach to generate original and/or multiple haploid reporter ESC lines in culture, since sgRNA targeting sequences and short HAs of the donor vectors are easily alterable. Although self-diploidisation is an important issue in mouse haploid experiments, a culture condition using the Wee1 inhibitor PD166285 was recently shown to stabilise the haploid state^30^. In conclusion, this CRISPR/Cas9 mediated knock-in system can be applied for studies involving genetically engineered haploid ESCs, such as forward genetic screening.

## Methods

### Animals

All animal care and experiments were carried out in accordance with the guidelines approved by the Animal Care and Use Committee of Graduate School of Medicine, Osaka University.

### Establishment and maintenance of mouse parthenogenetic haploid embryonic stem cells

MII oocytes were collected from superovulated B6D2F1 (C57BL/6 x DBA2 F1) female mice, and parthenogenetic haploid ESCs were established via activation by strontium chloride, as described previously^3^. The DNA content of ESC lines was analysed using FACS Aria II (BD Biosciences) after treatment with 10 μg/ml Hoechst 33342 for 30 minutes at 37 °C. The haploid 1n peaks were purified by periodic FACS sorting and were maintained on mitomycin-C treated MEFs^3^. Purification of haploid ESCs was performed at least four times before transfection of the reporter cassette.

### Cell culture

Diploid ESCs (J1 strain) were cultured in ES medium consisting of the Glasgow modified Eagle’s medium (GMEM, high glucose) supplemented with 15% foetal bovine serum (FBS) (JRH Biosciences), 1000 U/ml LIF, 1 mM MEM-NEAA, 1 mM sodium pyrubate, 0.1 mM 2-mercaptoethanol, 100 U/ml Penicillin, and 100 μg/ml Streptomycin. Haploid ESCs were cultured on inactivated MEFs in ES medium supplemented with 2i: 3 μM CHIR99021 and 1 μM PD0325901. HEK293T cells were cultured in Dulbecco’s modified Eagle’s medium (DMEM, high glucose) supplemented with 10% FBS.

### Construction of knock-in donor vectors

The Venus-SVpA and PGK-neoR-bGHpA fragments were PCR amplified from CSII-EF-Venus (kindly gifted by Dr. H. Miyoshi, Riken BRC, Japan) and pTNN_HQ683722^50^, respectively. These fragments were inserted into the pBluescript II SK(+) plasmid using the ligation high (TOYOBO) or in-Fusion HD cloning kit (Clonetech). The GSG linker-P2A sequence^32^ was ordered as oligonucleotides and inserted upstream of Venus, resulting in P2A-Venus-SVpA-PGK-neoR-bGHpA (pV-neoR). The hygroR fragment amplified from the pCAG-hygro plasmid by PCR and the annealed oligonucleotides for GSG linker-T2A were cloned downstream of Venus in pV-neo, resulting in P2A-Venus-T2A-hygroR-SVpA-PGK-neoR-bGHpA (pV-hygroR-neoR). Homologous arms (HAs) of each donor vector were PCR amplified from mouse genomic DNA using primers listed in Supplementary Table S3. One nucleotide of the right HA targeting *Actb* was altered to disrupt the PAM sequence and prevent repetitive cleavage by Cas9 nuclease. Both left and right HAs were inserted into pV-neoR or pV-hygroR-neoR, forming the final knock-in donor vectors.

### Preparation of the Cas9/Cas9n and sgRNA expression plasmids

The bicistronic expression vector pX330-U6-Chimeric_BB-CBh-hSpCas9 (pX330, Addgene plasmid # 42230) was used to express Cas9 and a sgRNA^14^. The Cas9-D10A nickase mutant vector (pX330-n) was created by site-directed mutagenesis of pX330 according to the protocol from Strategene. Targeting sequences of sgRNAs were designed using “Optimized CRISPR design” available at the Feng Zhang Laboratory website^20^. Sense and antisense oligonucleotides of each sgRNA targeting sequence were cloned into pX330 and pX330-n at the *Bbs*I site after annealing. The sgRNA targeting sequences are listed in Supplementary Table S1.

### The single strand annealing assay

The pCAG-EGxxFP plasmid was kindly gifted from Masahito Ikawa, Biken, Osaka University (available at addgene, plasmid # 50716)^34^. The genomic fragment containing the sgRNA targeting site was PCR amplified and inserted between the EGxxFP fragment using the *BamH*I and *EcoR*I sites. Four × 10^5^ HEK293T cells were transfected with 1 μg pCAG-EGxxFP plasmids and 1.5 μg pX330 or pX330-n plasmids using 15 μg polyethylenimine in six-well plates. A pair of 0.75 μg pX330-n plasmids was used for the double-nicking strategy. EGFP fluorescence was assessed by a fluorescence microscope (Olympus IX70) and FACS Aria II 48 hours after transfection. sgRNAs and PCR primers were listed in Supplementary Table S1 and Table S3 , respectively.

### Establishment of knock-in haploid and diploid ESC lines

Plasmids were transfected into haploid and diploid J1 ESCs using Lipofectamine 2000 (Invitrogen) according to the manufacturer’s instructions with the following modifications: incubated DNA-reagent mixture in Opti-MEM was mixed with singly suspended cells in culture medium (v/v = 1:1 ratio). The final concentration was 1.0 ng/μl donor vectors, 1.5 ng/μl pX330 or pX330-n, and 5 × 10^3^ cells/μl. In the case of double nicking, a pair of 0.75 ng/μl pX330-n plasmids was used. Transfected cells were incubated for 5 minutes and were plated onto 0.1% gelatin coated wells at a density of 2.5 to 20 × 10^3^ cells/cm^2^. G418 (300 μg/ml) or hygromycin (200 μg/ml) selection was initiated after 24 hours and continued for 8 days. Drug-resistant colonies were independently collected from each well and were sorted using FACS Aria II after staining with 10 ng/μl Hoechst 33342. Haploid 1n populations or haploid Venus-positive populations (in the case of targeting *Actb*) were separately re-plated on inactivated MEFs at a low density (~50 cells/cm^2^). Individual colonies were manually picked under the microscope and maintained as described above. Fluorescence was observed using a fluorescence microscope (Olympus IX70). Genomic DNA from each ESC line cultured without MEFs was used for sequencing analysis of the knock-in allele after PCR amplification.

### Neural differentiation and FACS analysis

Neural differentiation was induced using an adherent monolayer culture in the N2B27 medium^37^,^38^. Undifferentiated Hap-SV cells cultured without MEFs were plated onto 0.1% gelatin-coated culture dishes at a density of 5–10 × 10^3^ cells/cm^2^ in N2B27 medium, as described previously^37^. Fluorescence was observed under a fluorescence microscope (Olympus IX70). Differentiated Hap-SV cells were dissociated at day 9 and analysed using FACS Aria II with Hoechst 33342 staining. Serum-free embryoid body-like (SFEB) culture was also used for neural differentiation^39^. Undifferentiated Hap-SV cells were cultured in suspension at a density of 5 × 10^4^ cells/ml in the medium consisting of GMEM (high glucose), 10% knockout serum replacement (Gibco), 1 mM MEM-NEAA, 1 mM sodium pyrubate, 0.1 mM 2-mercaptoethanol, 100 U/ml Penicillin, and 100 μg/ml Streptomycin. Half of the medium was replaced every 2 to 3 days. Floating embryoid-like bodies were dissociated at 9 days after differentiation and used for FACS analysis after treatment with Hoechst 33342. Venus-positive and -negative populations were sorted using FACS and were applied for RT-qPCR analysis.

### RNA isolation and RT-qPCR analysis

RNA was extracted from cells using the RNeasy micro Kit (Qiagen) according to the manufacturer’s instructions. Complementary DNA was synthesised using the ThermoScript I kit (Invitrogen) with random hexamer primers. RT-qPCR was performed on an Applied Biosystems 7900HT machine using Fast Universal SYBR Green master mix (Applied Biosystems). Relative expression levels were normalised to *Gapdh* mRNA. Statistical significance was determined using Student’s *t*-test or Tukey’s multiple comparison analysis. Primers are shown in Supplementary Table S4.

### Immunofluorescence microscopy

Cells were washed three times in phosphate buffered saline (PBS), fixed in 4% paraformaldehyde in PBS for 15 min at room temperature, permeabilized in 0.3% Triton X-100 in PBS for 15 min at room temperature, and left in blocking solution (5% normal goat serum in PBS) for 1 hour at room temperature. Then, cells were incubated overnight at 4 °C with primary antibodies against Sox1 (1:500 dilution; ab87775, Abcam) or Nestin (1:200 dilution; ab27952, Abcam). Samples were washed three times in PBS and incubated for 1 hour with a secondary antibody, Alexa Fluor 568 Goat Anti-Rabbit IgG (A11036, MP), which was diluted 1:500 with blocking solution. DNA was counterstained with 1 μg/ml 4’,6-diamidino-2-phenylindole (DAPI). Fluorescence was detected and imaged using a fluorescence microscope (Olympus IX70)

### Western blot

Cells were washed twice in PBS, collected and lysed on ice for 30 min in the benzonase (Novagen) and lysis buffer (20 mM HEPES, 150 mM NaCl, 2.5 mM MgCl_2_, 0.1% NP-40, 1 mM DTT and cOmplete protease inhibitor cocktail). After EDTA was added for a final concentration of 2 mM, each cell extracts was boiled in SDS sample buffer (62.5 mM Tris-HCl, 700 mM 2-Mercaptoethanol, 2% sodium dodecyl sulfate, 5% sucrose, 0.01% bromophenol blue) at 96 °C for 5 min. Five μg total proteins of each samples were separated by SDS-PAGE and transferred to polyvinylidene difluoride (PVDF) membranes (Millipore). The membranes were blocked in 5% skim milk in TBST at room temperature for 1 hour and incubated overnight at 4 °C with the primary antibodies against Nestin (1:1000 dilution; ab27952, Abcam) or β-Actin (1:5000 dilution; A5441, Sigma). Immunoreactive bands were probed at room temperature for 1 hour with the horseradish peroxidase (HPR)-linked anti-mouse IgG (1:5000 dilution; NA9310V, GE healthcare) or HRP-linked anti-rabbit IgG (1:5000 dilution; NA9340V, GE healthcare). The protein bands were detected by ECL western blotting detection reagent (GE healthcare).

## Additional Information

**How to cite this article**: Kimura, Y. *et al*. CRISPR/Cas9-mediated reporter knock-in in mouse haploid embryonic stem cells. *Sci. Rep.* **5**, 10710; doi: 10.1038/srep10710 (2015).

## Supplementary Material

Supplementary Information

## Figures and Tables

**Table 1:** Knock-in efficiency with various combinations.

**Combination No.**	**Cas9 type**	**sgRNA**		**HA of donor vector**	**The Venus-positive ratios**	
		**target**	**targeting seq. (nt)**		**Haploid (%)**	**Diploid (%)**
1	Cas9	*Actb*	R(20)	Actb	18.0±1.2	8.2±0.6
2	Cas9	*Actb*	R(17)	Actb	21.2±0.9	10.3±0.3
3	Cas9n	*Actb*	L(20)+R(20)	Actb	30.6±1.5	14.3±0.4
4	Cas9n	*Actb*	L(20)+R(17)	Actb	31.1±1.3	14.4±0.6
5	Cas9n	*Actb*	R(20)	Actb	11.4±1.4	8.8±0.4
6	Cas9n	*Actb*	R(17)	Actb	4.0±0.5	3.3±0.2
7	Cas9n	*Sox1*	L(20)+R(17)	Actb	0.0±0.0	0.1±0.1
8	Cas9n	*Actb*	L(20)+R(17)	Sox1	0.1±0.1	0.0±0.0
9	No			Actb	0.0±0.0	0.0±0.0
					The ratio of knock-in ESC lines	
					Haploid (%)	
10	Cas9n	*Sox1*	L(20)+R(17)	Sox1	46.2 (n=26)	

The Venus-positive ratios were acquired using FACS 9 days after transfection and shown as the means ± SEM (n = 4). The knock-in efficiency at the Sox1 locus was calculated based on PCR analysis. A dot-plot graph and statistical analysis are shown in Fig. S5.
