# Supplementary material for: CRISPR/Cas9-mediated reporter knock-in in mouse haploid embryonic stem cells
Source: Sci Rep. 2015 Jun 3;5:10710. doi: 10.1038/srep10710 (PMC4454075; doi:10.1038/srep10710)
Supplement: Supplementary Information [file srep10710-s1.pdf]

Supplementary information

Title

CRISPR/Cas9-mediated reporter knock-in in mouse haploid embryonic stem cells

Authors

Yasuyoshi Kimura, Masaaki Oda, Tsunetoshi Nakatani, Yoichi Sekita, Asun Monfort, Anton Wutz, Hideki Mochizuki, Toru Nakano*

Corresponding author

*Toru Nakano

E-mail: tnakano@patho.med.osaka-u.ac.jp

Tel: +81-6-6879-3720; Fax: +81-6-6879-3729

Contents

Supplementary Figure S1-10

Supplementary Table S1-4

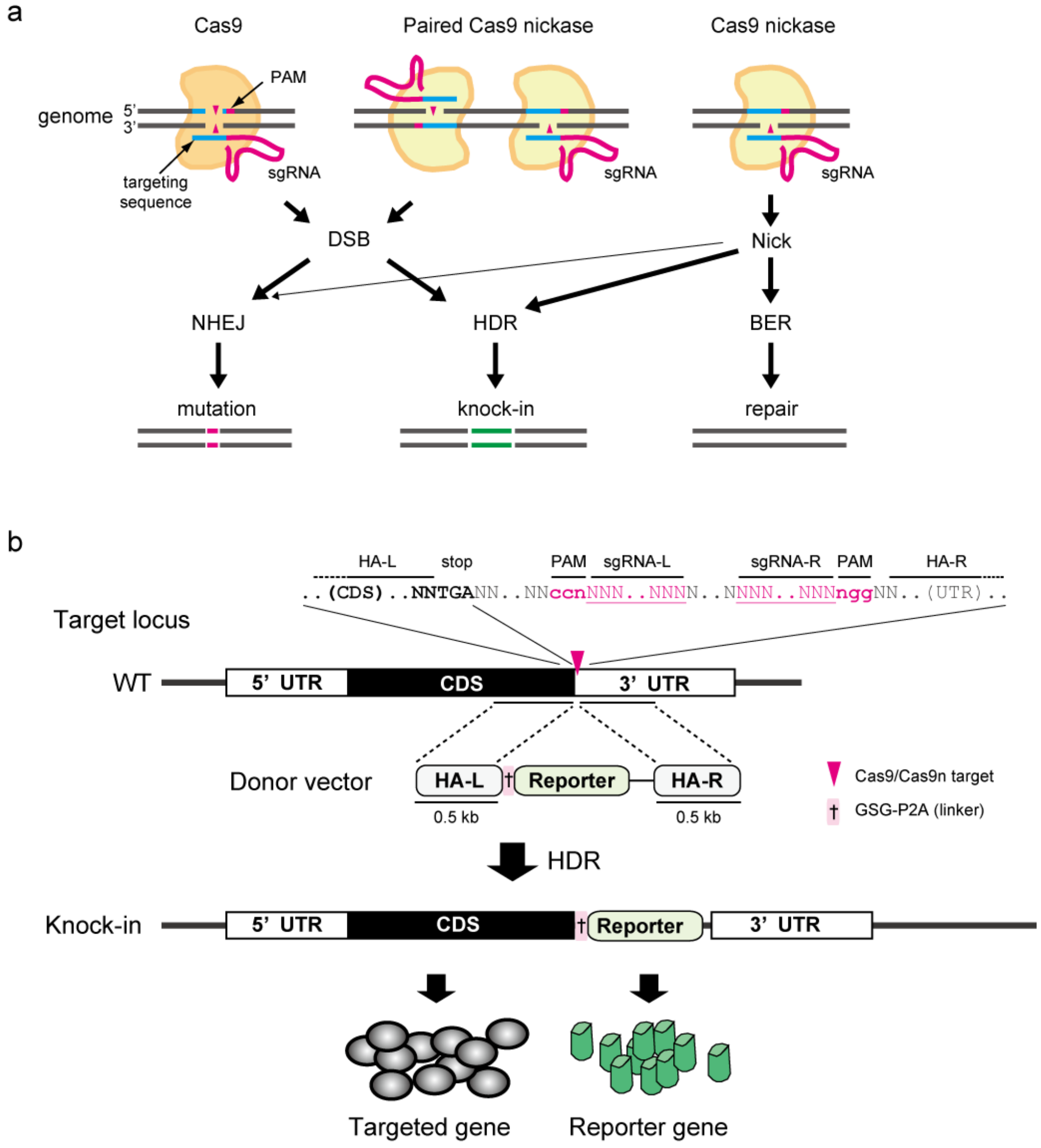

Supplementary Figure S1. CRISPR/Cas9-mediated genome engineering for the establishment of haploid reporter ESC lines

(a) Graphic representations of genome engineering using the CRISPR/Cas9 system. The Cas9 D10A mutant version is indicated as the Cas9 nickase. DSB, double-strand break; NHEJ, non-homologous end joining; HDR, homology dependent repair; BER, base excision repair. (b) Schematic illustration of the strategy to insert a reporter cassette into a target locus. The target locus and sequence representation for the CRISPR/Cas9 system are shown at the top. The CDS and its stop codon (e.g., TGA) are shown in black bold. The sgRNA for targeting sequences at left and right sides of the cleavage site (sgRNA-L and sgRNA-

R) are underlined and labelled in magenta. The PAM sequence is shown in magenta bold. The left and right homologous arms are indicated as HA-L and HA-R, respectively. The precise insertion via CRISPR/Cas9-mediated HDR fuses the reporter cassette and the last codon of the target gene by the self-cleaving peptide, P2A, leading to concordant expression of the target and reporter genes. PAM, protospacer adjacent motif; UTR, untranslated region; CDS, coding sequence.

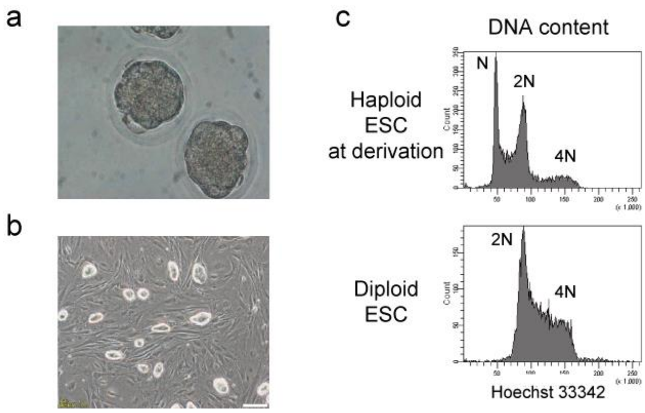

Supplementary Figure S2. Characterisation of mouse parthenogenetic haploid ESC lines

(a) Morphology of the activated embryos at the morula stage. (b) Morphology of ESCs established from parthenogenetic blastocysts. Scale bar, 200 µm. (c) DNA content analysed using FACS. The results of established haploid ESCs (top) and control diploid J1 ESCs (bottom) are shown.

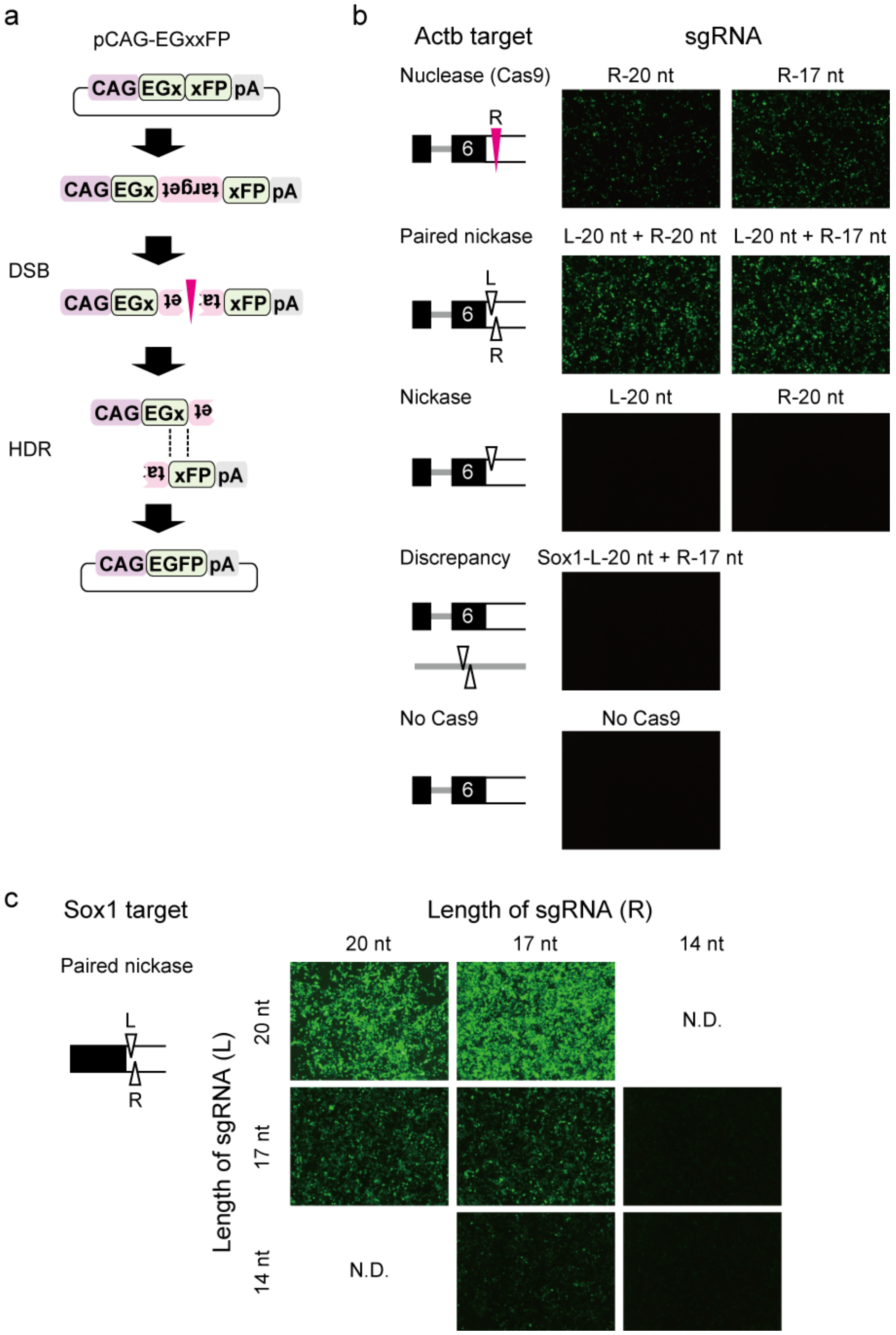

Supplementary Figure S3. Validation of sgRNA targeting sequences using the single-strand annealing assay

(a) Schematic overview of the strategy to validate on-target cleavage activity of designed sgRNAs using the pCAG-EGxxFP plasmid (the SSA assay)[34]. DSBs induced by the Cas9 or the paired Cas9n with

functional sgRNA(s) will lead to HDR and to reconstitute EGFP, whereas individual nicks by the Cas9n will not introduce HDR or yield fluorescence because the proper homology donor is absent in this case. (b, c) The excision efficiency at *Actb* (b) and *Sox1* (c) target sequences by the combination of Cas9 types and sgRNAs. Left diagrams illustrate the type of Cas9/Cas9n and right panels show fluorescence images of HEK293T cells 48 hours after co-transfection of the pCAG-EGxxFP plasmids and the Cas9/Cas9n-expressing plasmids. Magenta arrowhead represents Cas9 and white arrowheads represent Cas9n. L and R indicate left and right sgRNA targeting sequences, which are listed in Supplementary Table S1. The length of each sgRNA targeting sequence is also indicated. N.D., not determined.

a

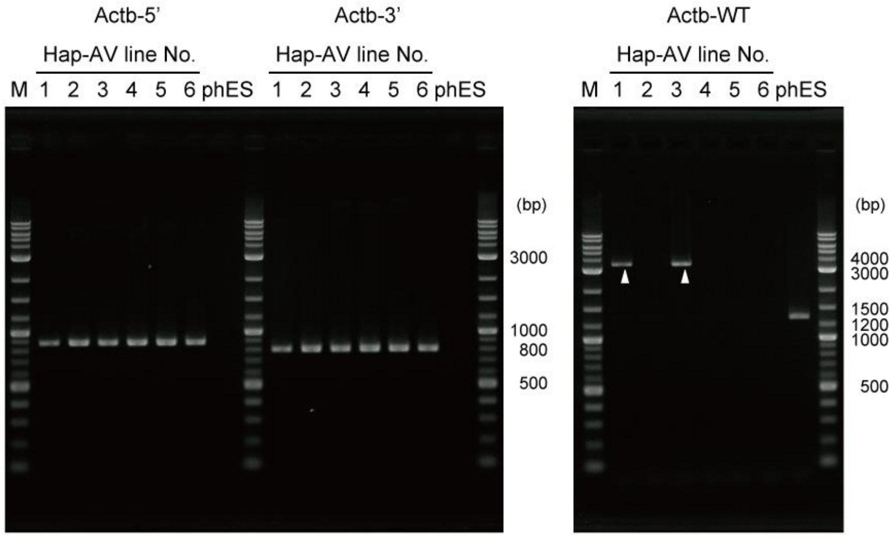

b

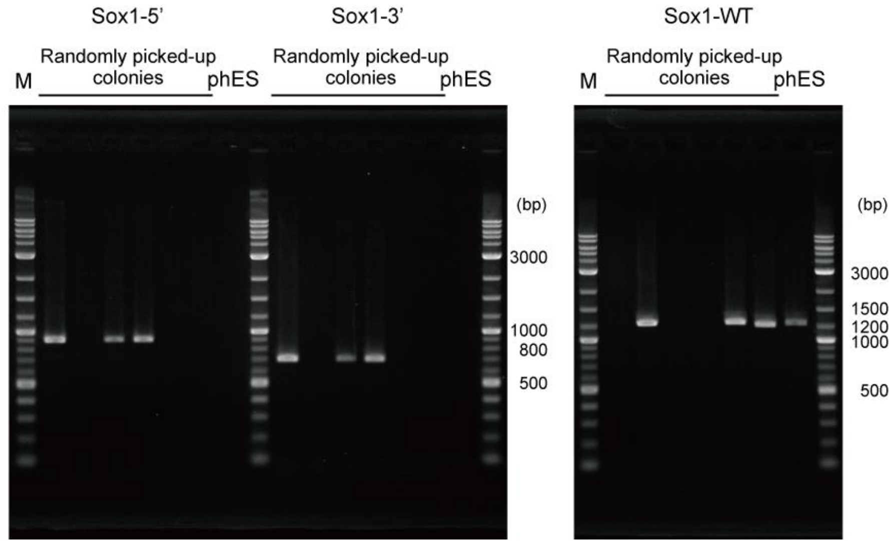

Supplementary Figure S4. Targeted knock-in of the reporter cassette detected by genomic PCR

(a) Full length electrophoretic gel images of genomic PCR in the Hap-AV lines. Representative 6 Hap-AV lines (No. 1-6) and their parental haploid ESC line (phES) are shown. The bands indicated by arrowhead may represent a knock-in long PCR product. Also refer to Fig. 1f. M, 2-Log DNA ladder marker. (b) Full length electrophoretic gel images of genomic PCR in randomly selected neomycin-resistant haploid colonies in the process of the establishment of Hap-SV lines. Their parental haploid ESC line (phES) is shown as a control. Also refer to Fig. 2b.

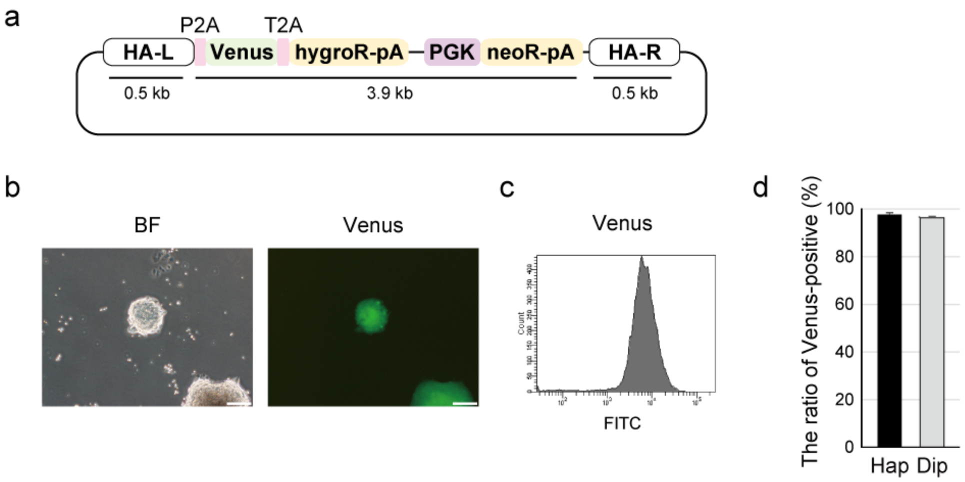

Supplementary Figure S5. Knock-in of the P2A-Venus-T2A-hygroR reporter cassette at the *Actb* locus

(a) The structure of a P2A-Venus-T2A-hygroR reporter cassette. (b) Morphology and Venus fluorescence images of the haploid ESCs carrying the P2A-Venus-T2A-hygroR reporter cassette isolated under hygromycin selection. BF, bright field; Scale bar, 200 µm. (c) Venus fluorescence of the haploid ESCs carrying the P2A-Venus-T2A-hygroR reporter cassette analysed by FACS. (d) The Venus-positive ratio of haploid and diploid ESCs after knock-in of the P2A-Venus-T2A-hygroR reporter cassette. Data are shown as the means ± SEM (n=3).

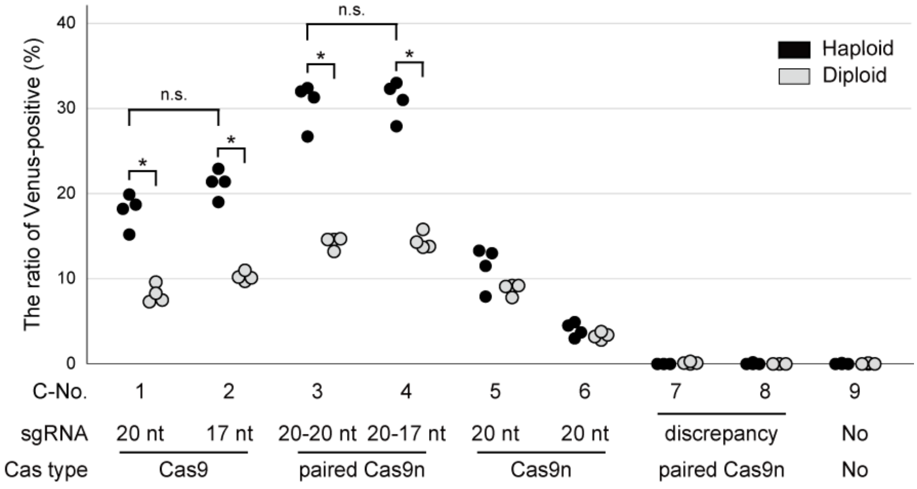

Supplementary Figure S6. Knock-in efficiency at the *Actb* locus

A dot-plot graph showing the Venus-positive ratio after CRISPR/Cas9-mediated knock-in of the P2A-Venus reporter at the *Actb* under several different conditions. Combination numbers (C-No.) below the graph correspond to the numbers in Table 1. Briefly, the length of sgRNA-targeting sequences and the transfected Cas9 type are described below each C-No. Discrepancy means that there was an inconsistency between the sgRNA target and the HAs of the donor vector. Data are shown as dot plots (n = 4). n.s., not significant when compared using Tukey's multiple comparison analysis at the 5% significance level in the haploid group. All other comparisons among C-Nos.1-6 in haploids indicated significant differences. *, p<0.01 by Student's *t*-test when comparing haploid to diploid in each C-No.

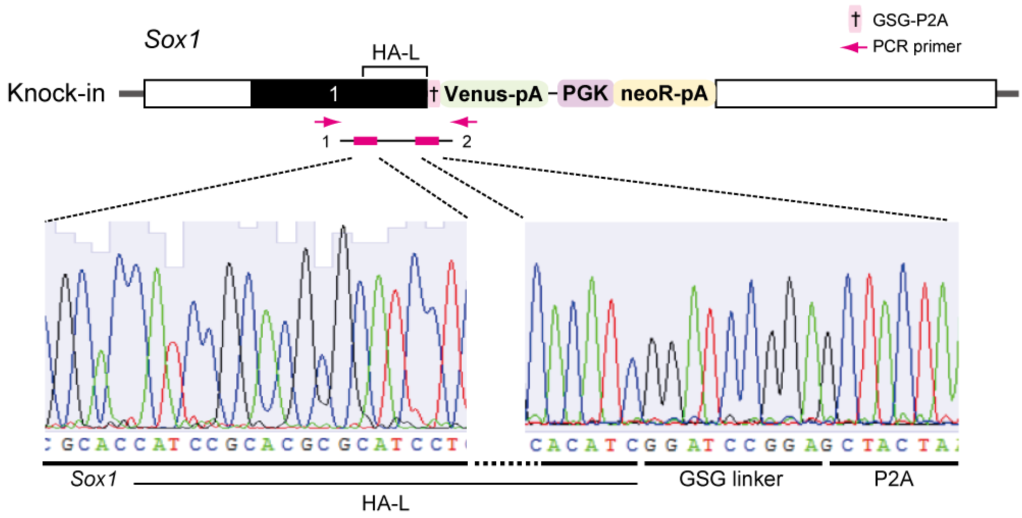

Supplementary Figure S7. Sequences of Hap-SV cells at the *Sox1* target site

The structure of the knock-in allele (top) and sequences around the left homologous arm (HA-L, bottom) in Hap-SV ESCs are shown. Sequences of parts of the PCR-amplified region (magenta box, middle) are shown as a representation.

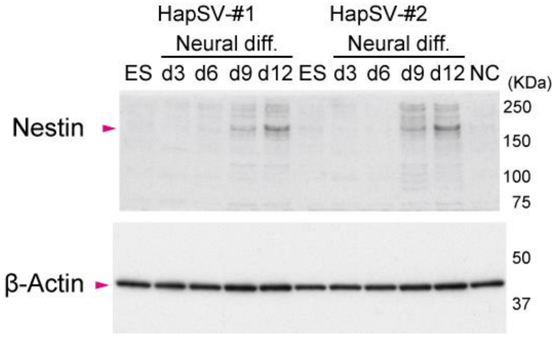

Supplementary Figure S8. Western blot analysis of Hap-SV cells along neural differentiation

Western blot analysis of early neural marker Nestin along neural differentiation in the N2B27 medium. Cells were collected at indicated time point and 5 μg total proteins were applied to each lane. Predicted band positions were indicated by magenta arrowhead. Neural diff., neural differentiation in N2B27 medium; NC, negative control (parent haploid ESCs cultured in ES medium).

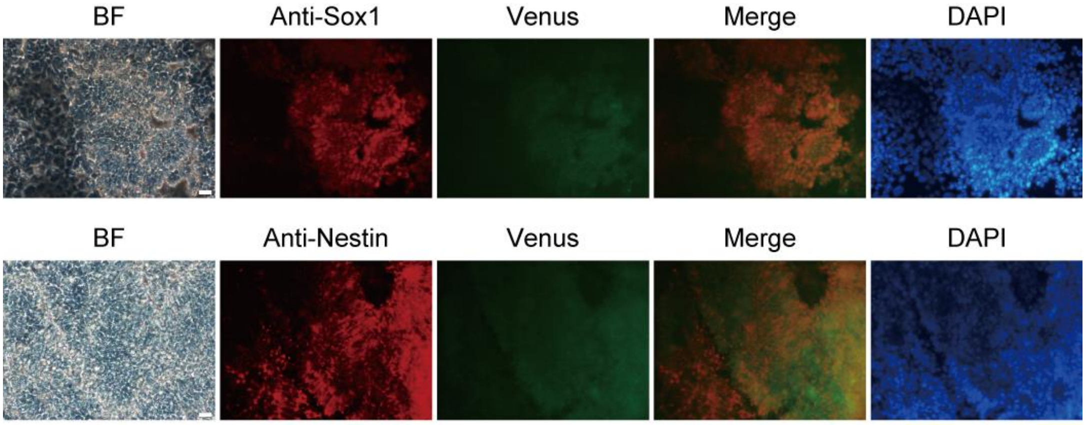

Supplementary Figure S9. Immunostained images of Hap-SV cells after neural differentiation

Immunostaining of Sox1 (top) and Nestin (bottom) in Hap-SV cells 10 days after neural differentiation. BF, bright field; scale bar, 20 µm.

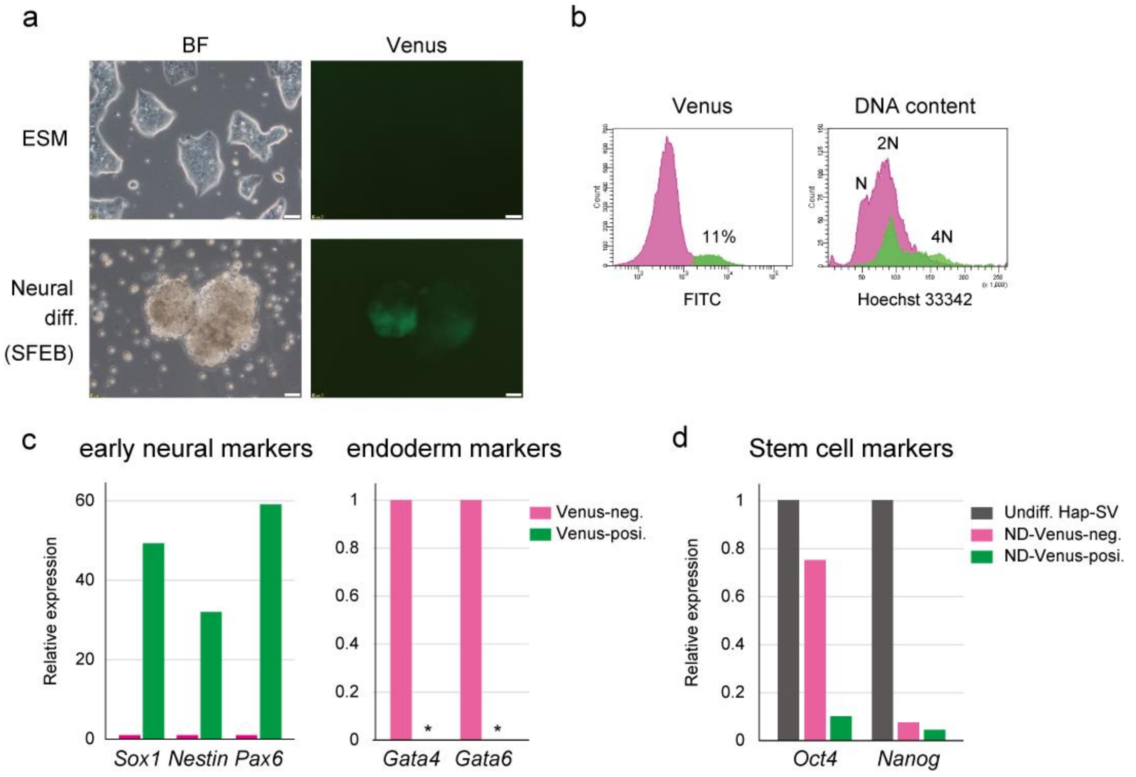

Supplementary Figure S10. Characterisation of neural cells differentiated from Hap-SV ESCs by the SFEB method

(a) Morphology and Venus fluorescence images of the Hap-SV ESCs (top) and neural cells 9 days after differentiation using the SFEB method (bottom). BF, bright field; Scale bar, 50 µm. (b) Venus fluorescence and DNA content analyses of the neural cells 9 days after differentiation from the Hap-SV cells. The Venus-negative population is shown in magenta and the Venus-positive population in green. (c, d) Expression of differentiation marker genes (c) and stem cell marker genes (d) in the differentiated Hap-SV cells. RT-qPCR was performed 9 days after differentiation using the SFEB method. Venus-negative (magenta) and Venus-positive (green) sorted populations were analysed. Relative expression levels were normalised to that of *Gapdh*. Values in Venus-negative populations (c) or in undifferentiated Hap-SV cells (d) were set to 1. *, not detected.

Supplementary Table S1 The parameter and cleavage activity of sgRNAs

Target	Name	sgRNA-targeting sequence	Length	GCs	Activity
Sox1	L	CAGTGTCCCCGGCGTCCCCG	20	16	+++
Sox1	R	CGCCCCGGCGACGAAGAGCG	20	16	+++
Sox1	R-g2	GCGGCTTAAGGCCGGCGCCC	20	16	+++
Actb	UTR-g2	GGTGTTGAGGCAGCCAGGGC	20	14	++
Sox1	UTR-g2	ATCTGCAGGGCCCAAGAGTA	20	11	++
Sox1	UTR-g3	GTTTAGCGTTTTAACTGCCA	20	8	+++
Nanog	T1	TATGAGACTTACGCAACATC	20	8	+++
Nanog	T2	ATGAGACTTACGCAACATCT	20	8	+++
Actb	R	AATAAGAGACAACATTGGCA	20	7	+
Actb	UTR-g1	TTTTAATTTCTGAATGGCCC	20	7	-
Actb	L	GTTAGGTTTTGTCAAAGAAA	20	6	+
Sox1	UTR-g1	TTTTAGATGAAAGACTTGCA	20	6	-
Nanog	T2-18 nt	GAGACTTACGCAACATCT	18	8	+++
Sox1	L-17 nt	TGTCCCCGGCGTCCCCG	17	14	-
Sox1	R-17 nt	CCCGGCGACGAAGAGCG	17	13	++
Nanog	T1-17 nt	GAGACTTACGCAACATC	17	8	+++
Actb	R-17 nt	AAGAGACAACATTGGCA	17	7	++
Sox1	L-14 nt	CCCCGGCGTCCCCG	14	13	-
Sox1	R-14 nt	GGCGACGAAGAGCG	14	10	-

The length, GC content and cleavage activity of each sgRNA with Cas9 examined using the SSA assay. sgRNA activity was evaluated using GFP fluorescence, as indicated below. The result is listed according to length and GC contents. Also refer to Supplementary Fig. S3 and Table S2.

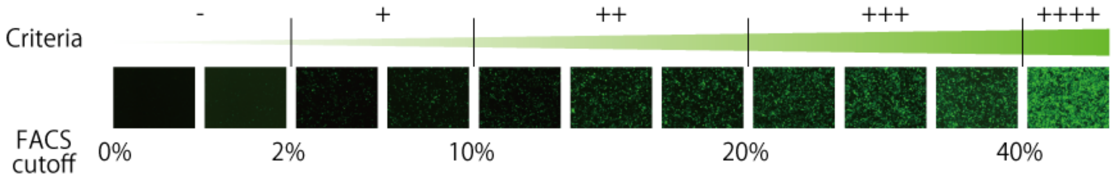

Supplementary Table S2 Double nicking and cleavage activity

Target	sgRNA name		sgRNA activity*		Activity**
	Left	Right	Left	Right	
Actb	L	R	+	+	++
	L	R-17 nt	+	++	++
Sox1	L	R-g2	+++	+++	+++
	L	R	+++	+++	++++
	L	R-17 nt	+++	++	++++
	L-17 nt	R	-	+++	++
	L-17 nt	R-17 nt	-	++	++
	L-17 nt	R-14 nt	-	-	-
	L-14 nt	R-17 nt	-	++	+
	L-14 nt	R-14 nt	-	-	-
Any	Any	-	various	none	-
	-	Any	none	various	-

Double nicking and cleavage activity with Cas9 nickases examined using the SSA assay. Cleavage activity was evaluated based on GFP fluorescence, as indicated below.
* individual sgRNA activity with Cas9 (Fig. S3); ** cleavage activity with the paired Cas9 nickase. Also refer to Supplementaru Fig. S3 and Table S1.

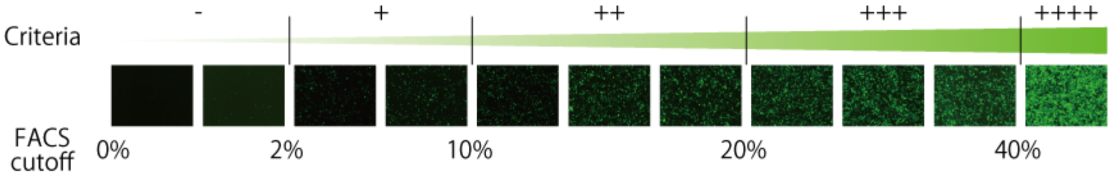

Supplementary table S3 The list of PCR primers

For the SSA assay	Sequence (5’ > 3’)
Actb-target-Forward	CGCGGGATCCTTAGCTTGGTGAGGGTG
Actb-target-Reverse	CGGAATTCGATGTTTGCTCCAACCAACTGC
Sox1-target-Forward	CGCGGGATCCTGCGCGAGATGATCAGCATG
Sox1-target-Reverse	CGGAATTCCGAAGCCGAGAAAACGCTGTG
**For amplifying HA**	**Sequence (5’ > 3’)**
Actb-HA-L-Forward	TCGAATTCGCGGATCGACAAAACTCCTGAGGCCATAG
Actb-HA-L-Reverse	TAGTAGCTCCGGATCCGAAGCACTTGCGGTGCACGATG
Actb-HA-R-Forward*	CTAGCTAGAGCTTAAGAGACAACATTGGCATAGCTTTG
Actb-HA-R-Reverse	CGGTGGCGGCCTTAACCTCAGACCTGGGCCATTCAG
Sox1-HA-L-Forward	TCGAATTCGCGGATCATCCGCACGCGCATCCTCACAAC
Sox1-HA-L-Reverse	TAGTAGCTCCGGATCCGATGTGCGTCAGGGGCACCGTG
Sox1-HA-R-Forward	CTAGCTAGAGCTTAACAGCCTCCAGAGCCCGACTTTG
Sox1-HA-R-Reverse	CCCGGCGACGAAGAGCG
**For PCR analysis of KI alleles**	**Sequence (5’ > 3’)**
Actb-1	GCAAGCTTGTAGCCTAGTCCTTTCTCCATC
Actb/Sox1-2	CGCGGAATTCGGGCATGGCGGACTTGAAG
Actb/Sox1-3	TCACTGCATTCTAGTTGTGGTTTGTC
Actb-4	TAGATACCATCAGCAAGGCCTAG
Sox1-1	CGAAGCTTCATGATGCAGGAGGCACAGCTG
Sox1-4	GCAGGCTATGTACAACATCCGAC

The list of PCR primers. Listed sequences include arms for ligation or in-Fusion if they have. * one nucleotide is altered for disrupting PAM sequence (red colored)

Supplementary table S4 RT-qPCR primers

Name	Sequence (5’ > 3’)
GAPDH-F	TGACCACAGTCCATGCCATC
GAPDH-R	GACGGACACATTGGGGGTAG
Oct4-F	CACGAGTGGAAAGCAACTCA
Oct4-R	AGATGGTGGTCTGGCTGAAC
Nanog-F	AACCAAAGGATGAAGTGCAAGCGG
Nanog-R	TCCAAGTTGGGTTGGTCCAAGTCT
Sox1-F	GGAAAACCCCAAGATGCACAAC
Sox1-R	CGCAGTCTCTTGGCCTCGTC
Nestin-F	AGGACCAGGTGCTTGAGAGA
Nestin-R	TTCGAGAGATTCGAGGGAGA
Pax6-F	GGGAAAGACTAGCAGCCAAA
Pax6-R	TGAAGCTGCTGCTGATAGGA
GATA4-F	TCTCACTATGGGCACAGCAG
GATA4-R	GCGATGTCTGAGTGACAGGA
GATA6-F	CAAAGCTTGCTCCGGTAAC
GATA6-R	TGAGGTGGTCGCTTGTGTAG
